# Food provision in daycare: perspective of daycare staff and parents in association with nutrient intake and dietary diversity of under-five children

**DOI:** 10.1017/jns.2026.10087

**Published:** 2026-04-30

**Authors:** Hanna Yuni Setiyaningrum, Rita Anggorowati, Umi Fahmida

**Affiliations:** 1 Department of Nutrition Science, Faculty of Medicine Universitas Indonesia − Dr. Cipto Mangunkusumo General Hospital, Jakarta, Indonesia; 2 Southeast Asian Ministers of Education Organization Regional Centre for Food and Nutrition (SEAMEO RECFON), Pusat Kajian Gizi Regional (PKGR) Universitas Indonesia, Jakarta, Indonesia; 3 Professional Teacher Education Program, School of Postgraduate Studies, Universitas Pendidikan Indonesia, Bandung, Indonesia; 4 Indonesian Centre for Educational Initiative, Bandung, Indonesia; 5 Early Childhood Care, Nutrition and Education (ECCNE) Working Group, Southeast Asian Ministers of Education Organization Regional Center for Food and Nutrition (SEAMEO RECFON), Jakarta, Indonesia

**Keywords:** Daycare, Dietary diversity, Food provision, Nutrient intakes, Shared values

## Abstract

This study aims to compare dietary diversity score (DDS), nutrient intakes and child feeding practices between under-five children who are provided meals by daycare (PM) or bring a lunchbox (LB) and to explore the perspective of mothers, daycare staff and management on their role in providing balanced and nutritious meals for under-five children. The cross-sectional study used mixed method. The quantitative part assessed nutrient intakes using 2-day non-consecutive dietary records and child feeding practices using Child Feeding Practices Questionnaire (CFPQ). Qualitative research employed WeValue Insitu and Perspective Exploration. The study was conducted at daycares in Ministries/Agencies in Jakarta, Indonesia, involving 138 children (6–59 months) and 124 working mothers. The qualitative sample comprised three working mothers, five daycare staff, and five daycare managers. Although DDS was higher among children aged 6–23 months in the PM group, energy and nutrient intakes were lower, with higher inadequacy of folate and calcium. Mothers in the LB had higher scores in environment, encouragement of balance and variety, and restriction subscales. Qualitative data identified role of daycare in providing meals or advice on lunchbox menus, skipping breakfast, eating environment between home and daycare, and nutrition knowledge. The higher nutrient intakes in LB were concurrent with better child feeding practices. Food provision in daycare, if not accompanied by nutrition education for parents, may lead to parents over reliance to daycare. Nutrition education is needed to enhance attitudes and awareness of working mothers and daycare staff regarding breakfast consumption, menu planning, child feeding practices, and portion sizes.

## Introduction

With the rise of urbanisation, the demand for daycare services has significantly increased as working mothers juggle various responsibilities, leading to a growing reliance on these facilities.^(1)^ Children enrolled in daycare can spend up to five 8-hour days each week.^(2)^ Data from the 2018 National Socioeconomic Survey (SUSENAS) indicate that daycare centres enrolment among Indonesian children under-five is very low, with only 0.79% attending daycare.^(3)^ Despite this limited coverage, understanding the nutritional environment within daycare settings is still crucial as children in full-time daycare are recommended to consume one-half to two-thirds of his or her daily dietary intake at daycare, indicating the importance of daycare for children’s dietary intake.^(4)^

One notable aspect of daycare is the significant variation in the provision of meals.^(2)^ While some daycares provide meals for the children, daycare that do not provide meals for children will rely on children to bring their own from home (a lunchbox).^(2,5)^ The rationale behind this disparity can involve multifaced perspectives on the importance of providing meals to children in daycare.^(6)^ Studies showed that the children’s lunchbox content do not meet balanced diet and are typically unhealthy, lacking in fruits and vegetables, and often containing sugar-sweetened beverages and snacks.^(7–10)^ In contrast, daycare-provided meals have been shown to improve children’s diets and have better nutrient profiles than meals consumed at home.^(11)^ For example, a study among Finnish children found that diet of children in daycare was more balanced and closer to dietary recommendations compared to children cared for at home.^(12)^

The difference in dietary intake between children who are provided with meals and those who are not will have an impact on their nutrient intakes and dietary diversity. Dietary diversity is a good predictor of dietary quality and micronutrient density.^(13)^ Lack of dietary diversity is closely related to poor diet quality, which indicates consumption of a low variety food.^(14)^ This condition has been associated with inadequate nutrient intakes that can lead to malnutrition in under-five years children.^(15)^ National data from Indonesia support this concern, showing that 55.7% of under-five years children consumed less than 80% of the energy Recommended Dietary Allowance (RDA), and 23.6% had inadequate protein intake^(16)^ Micronutrient inadequacies were also widespread, especially for iron, zinc, calcium, and vitamin C, highlighting the importance of improving dietary diversity and increasing the intake of nutrient among children in this age group.^(17)^

Previous study in Netherlands that explore facilitators and barriers to healthy nutrition and physical activity in childcare from different perspectives (childcare managers, childcare workers and parents) found that all respondents expressed a positive attitude towards healthy nutrition and physical activity in childcare.^(18)^ Mothers, as primary caregivers, may have distinct preferences and practices when it comes to preparing and packing their child’s meals.^(19)^ On the other hand, staff and daycare manager play a vital role in menu planning, food preparation, and the overall food environment within the daycare facility.^(20,21)^ This finding suggest the importance of understanding values and perspective from the working mothers, daycare staff, and manager in providing meals for children.

Therefore, this study aims (1) to compare nutrient intakes in under-five children, and dietary diversity and child feeding practices in under-two children, between those who are provided meals by daycare (PM) and those who brought a lunchbox (LB); and (2) to explore the perspective of mothers, staff, and daycare managers related to their role in providing balanced and nutritious meals for under-five children. The results of this study are expected to serve as a foundation for strengthening education for mothers, staff, and daycare manager in providing balanced and nutritious meals to under-five children.

## Methods

### Study design and study area

This mixed-method study was part of the baseline of the Early Childhood Care, Nutrition and Education (ECCNE) Daycare project. Quantitative (comparative cross-sectional) and qualitative (shared values) data were collected at 11 daycares in Ministries/Agencies in Jakarta, Indonesia, from November 2023 to January 2024.

This study was conducted according to the guidelines laid down in the Declaration of Helsinki and all procedures involving research study participants were approved by the Ethical Review Committee, Faculty of Medicine, Universitas Indonesia (Reference Number: KET-1540/UN2.F1/ETIK/PPM.00.02/2023). Written informed consent was obtained from parents.

### Study population

The study included children aged 6–59 months enrolled in daycare facilities at 11 Ministries/Agencies in Jakarta. This analysis used baseline data from an intervention study and was treated as a cross-sectional comparison. The minimum sample size for the baseline analysis was calculated using G*Power for hypothesis testing comparing two independent means (one-sided test), aiming to compare nutrient intake between children who received daycare-provided meals and those who brought lunchboxes. The calculation was based on available data for energy intake, assuming 80% power, a 5% significance level, and allowing for a 10% dropout/non-response rate. The minimum required sample size was 28 children per group. The final sample size was larger (60 per group), as it followed the requirement of the parent intervention study, which was calculated based on an expected difference of 10 points in the BSID-III socio-emotional score, a standard deviation of 14, 95% power, a design effect of 2, and a 10% dropout rate.^(22)^

For qualitative data collection, daycare staff, managers, and working mothers were recruited for the WeValue Workshops in the ECCNE project, focusing on Shared Values and Perspective Exploration (PEX). Purposive sampling targeted participants with relevant expertise. Thirteen respondents participated which consist of working mothers (*n* = 3), daycare staff (*n* = 5), and daycare managers (*n* = 5).

### Data collection

#### Quantitative data collection

##### Socio-demographic assessment

Socio-demographic characteristics, including child’s sex and age, maternal age, marital status, household income, maternal education, and family size, were collected using a structured questionnaire. Staff characteristics such as age, education level, and employment length were also assessed for the WeValue workshops.

##### Anthropometry assessment

Anthropometric measurements were carried out by trained enumerators and included assessments body weight and length (for children below 24 months) or height (for children aged 24 months and older). Weight was measured with a SECA scale (0.1 kg precision), while length/height was measured with a shorrboard (0.1 cm precision).

##### Dietary assessment

Dietary intake was assessed using a combination of estimated and weighed records over two non-consecutive days, covering weekdays and weekends. At daycare, weighed records were used when children were present, supplemented by mothers’ records of meals consumed at home or when children were away. Daycare staff were trained by enumerators on the procedure and supervised meal weighing. Food items, including portion sizes, brand names, and preparation methods, were recorded. Left-over portions were weighed to determine actual intake. Working mothers were trained by enumerators on the dietary record procedure and its completion. Dietary records for two days were completed by working mothers, and included meals consumed at home or elsewhere, specifying mealtimes, locations, and ingredients including brand names. Standardised measures, food photographs, and food models were used to aid in estimating portions and recording recipes. Dietary records of the children, as completed by mothers, were reviewed by enumerators during subsequent visits at daycare.

Food consumption data were analysed using the Kobo Toolbox application to convert into nutrient intakes. Energy intake for breastfeeding infants and young children was assessed against acceptable ranges based on WHO/UNICEF, with adjustments made based on breastfeeding energy contributions (413, 379, and 346 kcal/day for ages 6–8 months, 9–11 months, and 12–23 months, respectively).^(23)^

The assessment of energy underreporting and overreporting for non-breastfeeding was calculated using the McCrory formula.^(24)^ The equations for predicted total energy expenditure (pTEE) are as follows:

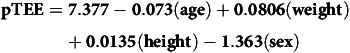




Where age is in years, weight is in kg, height is in cm, and sex is 0 for male and 1 for females. Under reporters were identified as those with energy intake/pTEE value of less than 40%, while over reporters were those with an energy intake/pTEE value greater than 160%.

##### Minimum Dietary Diversity (MDD) assessment

Minimum Dietary Diversity (MDD) in this study was determined based on the dietary diversity score (DDS) of children aged 6–23 months. It assessed whether children consumed foods and beverages from at least five out of eight specified food groups within the previous day. The food groups included: (i) Breast Milk; (ii) Grains, white roots, and tubers; (iii) Legumes, nuts, and seeds; (iv) Dairy products (milk, yogurt, cheese); (v) Flesh foods (meat, fish, poultry, liver/organ meats, seafood); (vi) Eggs; (vii) Vitamin A-rich fruits and vegetables; and (viii) Other fruits and vegetables.^(25,26)^

##### Child feeding practice assessment

This questionnaire was adapted from the validated Child Feeding Practices Questionnaire (CFPQ)^(27)^ and included a total of 32 questions related to child feeding practices, suitable for daycare settings. The 32 items of the CFPQ were categorised into eight feeding practice subscales, including child control (5 items), pressure to eat (5 items), teaching about nutrition (3 items), emotion regulation (3 items), environment (4 items), encourage balance and variety (4 items), modelling (4 items), and restriction (4 items).

### Qualitative data collection

Following the quantitative data collection, qualitative data was gathered through Perspective Exploration (PEX) interviews, which was conducted as part of the WeValue Workshop, an approach designed to elicit values in a group context and connect them to concrete actions, feelings, or perceptions. Three workshop sessions were held with daycare staff (*n* = 5), daycare management (*n* = 5), and working mothers (*n* = 3). The workshops, lasted 1 to 2 hours each, aimed to gain a deeper understanding of the principles governing the provision of balanced and nutritious meals to children in daycare settings.

At the start of each session, the research team provided an overview of the study’s objectives and discussion scope. Informed consent was obtained to ensure voluntary participation. The WeValue workshop utilised the WeValue InSitu method, which included stages such as contextualisation, photo elicitation, trigger list, collective exploration group, and value framework construction.^(28,29)^

After completing the WeValue InSitu study, the PEX continued with FGDs to explore the perspectives of working mothers, daycare staff, and management on their roles in providing nutritious food for children. All interviews were recorded, translated, and transcribed for analysis. A triangulation process was also conducted, involving 7 working mothers, 1 staff member, and 1 management representative.

## Data analysis

### Quantitative data analysis

Data from the study were coded, entered, and analysed using IBM SPSS Statistics for Windows, version 25.0. Continuous variables underwent normality testing using the Kolmogorov–Smirnov and Shapiro–Wilk tests. Descriptive statistics included mean and standard deviation for normally distributed data, and median (25^th^–75^th^) for skewed distributions. Independent t-test/Mann Whitney were used to compare the median values of two groups with significance value at *p* < 0.05. Categorical variables were compared between PM and LB groups using the Chi-square test/Fisher Exact test.

Nutrient intake analysis was conducted for all children across different age groups (6–11 months, 1–3 years, and 4–6 years). Only participants with complete dietary data from two non-consecutive days, recorded by daycare staff and mothers, were included. The Indonesian food composition database was used to convert dietary to nutrient intake data, using KoboToolbox application. Average nutrient intake over two days was computed using MSM tools. Intake levels were compared with the Recommended Nutrient Intake (RNI) guidelines for Indonesia (*Angka Kecukupan Gizi, AKG*) for children aged 6–11 months, 1–3 years, and 4–6 years. Proportion of inadequate nutrient intake was assessed using specific cut-off points for children aged 1–3 years and 4–6 years. The dietary adequacy of each nutrient was determined by comparing nutrient levels with the Estimated Average Requirement (EAR) using MSM tools.^(30)^ For iron, a full probability approach was employed to assess the prevalence of inadequacy.^(31)^ The detail was shown in Table 1.

**Table 1.** Nutrient requirements and adequacy levels used in assessing dietary adequacy of the children

Nutrient	Conversion Factor	Children (1–3 years)		Children (4–6 years)	
		RNI^a^	Adequacy cut-off	RNI	Adequacy cut-off
Energy (Kcal)^b^	–	1350	EER	1400	EER
Protein (g)^c^	–	20	PER	25	PER
Calcium (mg)^d^	1.20	650	542	1000	833
Folate (mcg)^d^	1.25	160	128	200	160
Iron (mg)^e^	–	7	–	10	–

a Indonesian RNI 2019.

b Adequacy was based on Estimated Energy Requirement (EER) for each individual, calculated as 82.5*body weight (for 1–3year children) or 73*body weight (for 4–6yr children).

c Adequacy was based on Protein Energy Requirement (PER) for each individual, calculated as 1.03*body weight (for 1–3yr children) or 0.87*body weight (for 4–6yr children).

d Adequacy was based on Estimated Average Requirements (EAR) = Indonesia RNI/Conversion Factor.

e Adequacy was based on full probability approach.

The internal reliability of the CFPQ was assessed using Cronbach’s alpha, and the tool was considered to have internal consistency if Cronbach’s alpha was equal to or higher than 0.7. The same CFPQ were used for mothers and staff. After reliability testing was conducted, an independent *t*-test/Mann Whitney test was applied to determine the differences between working mothers whose children were provided meals by daycare and those who bring lunch boxes. A *p-value* of less than 0.05 was considered statistically significant.

### Qualitative data analysis

For PEX analysis, recordings from the focus group discussion were transcribed by researcher. Before proceeding with the analysis, the transcripts were checked to ensure their completeness and accuracy. Codes that emerged from transcripts of working mothers, daycare staff, and daycare managers were used as the main data to address the main research question which focused on their roles in providing food for children. Three levels of coding were applied to the PEX transcripts to identify emerging themes.

## Results

### Characteristics of children

Of the 147 children whose parents consented to participate, socio-demographic and dietary intake data were available from 138 children. Nine children were excluded from the analysis due to exceeding the age eligibility criterion (over 59 months).

Both PM and LB groups had a higher percentage of children aged 24–59 months than those aged 6–23 months. LB group had a significantly higher percentage of older children (75%) compared to PM group (57.4%) (Table 2). More mothers in LB group had postgraduate education than those in PM group.

**Table 2.** Characteristics of the children who were provided meals by daycare (PM) and children who brought a lunchbox (LB)

Characteristics	Total (*n* 138)		PM (*n* 94)		LB (*n* 44)		*P-Value* ^†^
	*n*	%	*n*	%	*n*	%	
Sex of the child							0.388
Boys	67	48.6	48	51.1	19	43.2	
Girls	71	51.4	46	48.9	25	56.8	
Age of the child (months)							0.046
6–23	51	37.0	40	42.6	11	25.0	
24–59	87	63.0	54	57.4	33	75.0	
Age of mothers (years)							0.051
<31 (24–30)	54	39.1	40	42.6	14	31.8	
31–40	77	55.8	52	55.3	25	56.8	
>40 (41–47)	7	5.10	2	2.10	5	11.4	
Marital status							–
Single	0	0	0	0	0	0	
Married	138	100	94	68.1	44	31.9	
Family income (IDR)^a^, *N* = 133							0.104
Low	33	24.8	17	18.9	16	37.2	
Low middle	41	30.8	28	31.1	13	30.2	
Upper middle	26	19.5	19	21.1	7	16.3	
High	33	24.8	26	28.9	7	16.3	
Maternal education							<0.001
Highschool	19	13.8	17	18.1	2	4.50	
Undergraduate	91	65.9	67	71.3	24	54.5	
Post-graduate	28	20.3	10	10.6	18	40.9	
Family size							0.173
1–4	107	77.5	76	80.9	31	70.5	
≥5	31	22.5	18	19.1	13	29.5	

IDR, Indonesian Rupiah.

† Chi-Square test.

a Low if the quartile score is <12,250.000; Low Middle if the quartile score ranges from 12,250.001–16,000.000; Upper Middle if the quartile score ranges from 16,000.001–20,900.000; High if the quartile score is >20,900.001.

### Minimum dietary diversity amongst children 6–23 months

Out of the 138 children enrolled in the study, 46 children aged 6–23 months were selected for DDS analysis, consisting of 39 children in PM group and 7 children in LB group. There was a higher median DDS in PM group (7.0) compared to LB group (5.0), with PM group scores ranging from 5.0 to 8.0 and LB group scores from 5.0 to 6.0 (Table 3). All children in both groups (100%) met the MDD cut-off (≥5 food groups), with consistent consumption of grains, white roots, and tubers. PM group significantly consumed more eggs (87.2%) compared to LB group (28.6%).

**Table 3.** Distribution (%) of dietary diversity scores (DDS) and food groups consumed by children 6–23 months who were provided meals by daycare (PM) and children who brought a lunchbox (LB)

	PM (*n* 39)		LB (*n* 7)		*P-value* ^†^
	n	%	n	%	
**Dietary diversity score, median (min–max)**	7.0 (5.0–8.0)		5.0 (5.0–6.0)		0.004
MDD <5(Inadequate)	0	0	0	0	–
MDD ≥5(Adequate)	39	100	7	100	
**Food groups**					
Breast milk	30	76.9	6	85.7	1.000
Grains, white roots, and tubers	39	100	7	100	–
Legumes, nuts, and seeds	28	71.8	3	42.9	0.193
Dairy products (milk, yogurt, cheese)	29	74.4	3	42.9	0.176
Flesh foods (meat, fish, poultry and liver/organ meats, sea foods)	32	82.1	6	85.7	1.000
Eggs	34	87.2	2	28.6	0.030
Vitamin A-rich fruits and vegetables	35	89.7	5	71.4	0.221
Other fruits and vegetables	32	82.1	6	85.7	1.000

RNI, Recommended Nutrient Intake.

† Chi-square test/Fisher exact test; Mann–Whitney test for dietary diversity score.

#### Qualitative: The role of daycare in providing meals or advice on lunchbox menus

The qualitative findings confirmed the quantitative results. Both staff and management at the PM daycare noted that the menu was varied and changed regularly on a five-day cycle, offering options like chicken, beef, and eggs to provide a balanced intake of protein and vegetables.

*“* ***It’s scheduled….,*** *everything changes regularly…* ***so within 5 days, there’s already chicken, beef, eggs… because they’ve already prepared it*** *.” (Staff from PM group, primary school education, 7 years of work)*

*“For the menu, we have made efforts to include protein and vegetables* **. *We try to ensure that there’s chicken, fish twice a week, eggs, and sometimes we combine with some meat.*** *We have varied the fruits…. We alternate them over five days.” (Management from PM group, diploma education, 21 years of work).*

The daycare incorporated feedback from mothers and the school principal when providing meals. The principal created balanced, nutritious menus by consulting nutritionists, drawing inspiration from social media, and conducting online searches.

*“…. yesterday,* ***I consulted a nutritionist.*** *Because, well, it turns out it is difficult to find menus… because if we* ***search on Instagram***, *there are indeed instant meal options, different from those that really have vegetables, protein, vitamins, balanced like that.” (Management from PM group, diploma education, 21 years of work).*

The statements from daycare staff and management match those of the mothers. Working mothers also noted the menu’s diversity, which was documented in the communication book.

*“The teacher* ***writes it in the communication book,*** *what food was served. I would* ***say it is quite varied***, *very varied actually…” (Working mother from PM group, post-graduate education)*

Staff and management guided and suggested changes to children’s lunchbox contents, addressing concern about monotonous meals and advising parents if children seemed bored with their food.

*“…..* ***I have often given instructions,*** *if possible, the menu should be changed regularly, not the same every time. The important thing is variety, there should be vegetables, main dishes, and fruits” (Management from LB group, bachelor education, 8 years of work)*

*“Yes, sometimes* ***we provide information or suggestions***, *like “try bringing this tomorrow” or “try using this.” Often, children prefer advice from daycare teachers over their parents because they are busy working.” (Staff from LB group, high school education, 25 years of work)*

### Nutrient intake and proportion of inadequate nutrient intakes

#### Quantitative

A total of 113 children had the two-day dietary records completed. However, the energy intake of one child was identified as underreported and was subsequently excluded from the analysis. Thus, the number of children to be analysed is 112, with 77 in PM group and 35 children in LB group. The study found significant differences in protein intake between 6–11mo infants in the PM and LB groups (*P* < 0.05), with higher intake in the LB group (Table 4). Among children aged 1–3 years and children aged 4–6 years, no significant differences were found in energy, macronutrient, and micronutrient intake between PM and LB groups. There was no significant difference in median nutrient intakes between the PM and LB groups (Table 4).

**Table 4.** Daily intakes of energy, macronutrients, and micronutrients of children who were provided meals by daycare (PM) and wchildren who brought a lunchbox (LB)

Nutrient intake	Age category	RNI (EAR)	Total			PM			LB			*P*-Value^†^
			*N*	Mean	SD	*N*	Mean	SD	*N*	Mean	SD	
Energy (Kcal)	6–11 months	800	16	614	263	11	522	166	5	817	338	0.096
	1–3 years^a^	1350	51	1160	1040–1308	38	1147	926–1318	13	1168	1106–1255	0.538
	4–6 years	1400	45	1408	188	28	1400	181	17	1421	203	0.616
Protein (g)	6–11 months	15	16	26.0	10.0	11	21.9	6.20	5	31.1	11.5	0.034
	1–3 years	20	51	41.1	9.00	38	40.5	9.20	13	42.8	8.40	0.810
	4–6 years	25	45	50.1	9.00	28	51.4	9.30	17	48.0	8.50	0.321
Fat (g)	6–11 months	35	16	25.6	8.70	11	23.6	7.80	5	30.0	9.70	0.730
	1–3 years	45	51	40.1	9.20	38	40.4	10.3	13	39.0	4.70	0.609
	4–6 years^a^	50	45	43.1	38.4–52.9	28	41.0	37.3–53.1	17	46.9	40.9–52.4	0.266
Carbohydrate (g)	6–11 months	105	16	77.3	41.5	11	63.7	28.9	5	107	52.1	0.062
	1–3 years	215	51	147	35.8	38	142	38.4	13	163	20.3	0.180
	4–6 years	220	45	191	31.6	28	189	23.6	17	194	42.4	0.670
Calcium (mg)	6–11 months^a^	270	16	341	199–468	11	300	167–385	5	385	341–855	0.100
	1–3 years^a^	650 (542)	51	512	319–665	38	510	287–671	13	545	431–677	0.331
	4–6 years^a^	1000 (833)	45	612	528–796	28	590	546–764	17	650	461–826	0.870
Folate (mcg)	6–11 months^a^	80	16	84.4	36.7–512	11	68.5	36.7–513	5	114	48.3–137	0.193
	1–3 years^a^	160 (128)	51	128	100.2–154.6	38	126	100–156	13	137	89.3–159	0.914
	4–6 years^a^	200 (160)	45	134	108.5–165.9	28	134	108–163	17	148	110–175	0.963
Iron (mg)	6–11 months	11	16	7.70	4.30	11	6.90	4.60	5	9.50	3.50	0.120
	1–3 years	7	51	9.80	2.40	38	9.80	2.60	13	9.80	1.70	0.869
	4–6 years	10	45	11.3	2.80	28	11.3	2.80	17	11.3	3.00	0.903

RNI, Recommended Nutrient Intake.

EAR, Estimated Average Requirements.

a Median (25^th^–75^th^).

† Independent *T*-test; Mann–Whitney test.

**Figure 1.**
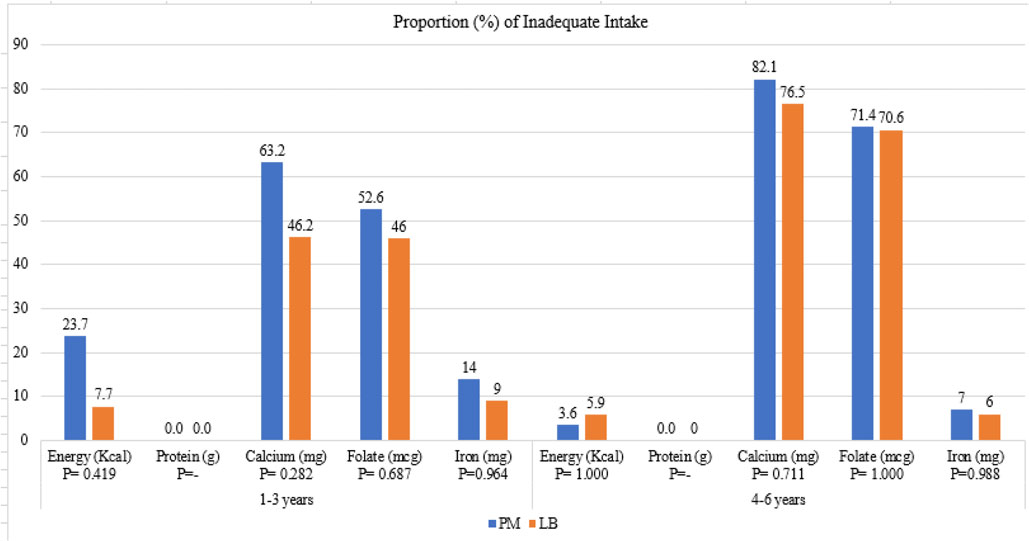
Proportion (%) of energy and nutrient intake inadequacy among children aged 1–3 years (*n* = 51) and children aged 4–6 years (*n* = 45) who were provided meals by daycare (PM) and children who brought a lunchbox (LB).

Figure 1 compared the proportion of children at risk of inadequate intake of nutrients in the PM and LB groups among ages 1–3 years and 4–6 years. For ages 1–3, no significant differences were observed between PM and LB groups in the risk of inadequate intakes of energy, calcium, folate, and iron. Nearly one in four children in the PM group (23.7%) had inadequate energy intake, while over 40% of children in both groups exhibited insufficient calcium and folate intake. For ages 4–6, no significant differences were found between PM and LB groups in the risk of inadequate intake of energy, calcium, folate, and iron. Overall, inadequate calcium and folate intakes were higher in children aged 4–6 years compared to those aged 1–3 years.

### Child feeding practices

#### Quantitative

Table 5 provided information regarding the differences in child feeding practices between working mothers in the PM group and the LB group. Overall, the scores in each feeding practice category indicated that working mothers in the LB group had higher scores compared to working mothers in the PM group. The scores of working mothers in the LB group were higher than those in the PM group, with significant differences (*p* < 0.05) found in the categories of environment (4.00 vs 3.75), encouragement of balance and variety (4.62 vs 4.25), and restriction (3.70 vs 3.40).

**Table 5.** Differences of child feeding practice between working mothers whose children were provided meals by daycare (PM) or brought a lunchbox (LB)

Category feeding practice	Working mothers in PM (*n* 92)		Working mothers in LB (*n* 32)		*P-value* ^†^
	Median	(25^th^–75^th^)	Median	(25^th^–75^th^)	
Child control	3.20	1.00–5.00	3.30	1.00–4.80	0.128
Pressure to eat	3.50	1.00–5.00	3.50	1.50–5.00	0.508
Teaching about nutrition	4.00	1.50–5.00	4.50	2.00–5.00	0.270
Emotion regulation	2.00	1.00–4.00	2.00	1.00–5.00	0.890
Environment	3.75	2.25–4.75	4.00	2.50–4.50	0.010
Encourage balance and variety	4.25	2.75–5.00	4.62	2.50–5.00	0.044
Modelling	4.25	1.75–5.00	4.62	2.75–5.00	0.371
Restriction	3.40	1.80–5.00	3.70	1.60–5.00	0.036

† Independent T-test; Mann–Whitney test.

#### Qualitative: Skipping breakfast

The qualitative findings revealed that daycares in the PM group did not provide breakfast, thus the meals started only at morning snack or lunchtime. Staff noted frequent hunger complaints from children, and parents occasionally confirmed their children had not been fed before arriving.

*“* ***They do not have breakfast***, *Ma’am, they are just given biscuits. Parents expect that at 10 AM, the children will get fruit and at lunch, they will get a snack” (Staff from PM group, high school education, 5 years of work).*

*“Sometimes, parents say,* ***‘My child/the child hasn’t breakfast yet, so let them eat from daycare, yeah?*** *‘” (Management from PM group, diploma education, 21 years of work).*

The daycare management did not provide breakfast facilities or feeding services, requiring children to eat breakfast at home. Working mothers confirmed this situation and explained that due to time constraints, they sometimes skipped providing breakfast and relied on daycare food, indicating limited awareness

*“At the daycare,* ***there was no breakfast service provided, and children were not allowed to bring packed lunche*** ***s**, so they had breakfast at home before coming to daycare. Here, we do not have a feeding service… So, if someone had not eaten breakfast yet, they would eat later according to the schedule, and they would end up eating a lot during lunchtime.” (Management from PM group, diploma education, 21 years of work).*

*“….* ***Sometimes I do not have time to give breakfast to my child/the child*** *before going to the daycare because the time is limited, so the child has not had time to have breakfast and I think it will be fed at the day care later at the morning snack and lunch” (Working mother from PM group, bachelor education)*

##### Qualitative: The eating environment between home and daycare

In line with lower CFPQ in encouraging balance and variety, a mother in LB group mentioned that she provided a complete meal in their child’s menu. On the other hand, a mother in the PM group mentioned that there were limitations in providing a variety of foods at home, and she rarely introduced variety foods to their child.

*“Certainly… what is mandatory is the animal-based ones, while the plant-based ones are more occasional, like tempeh or tofu…* ***The packed lunch includes and home meals a complete menu:*** *rice, dishes (chicken, meat, fish, eggs), vegetables, and fruit. (Working mother from LB group, post graduate education)*

*“Maybe at home,* ***we kind of have limitations in preparing a variety of foods,*** *Ma‘am. But if I think about it, we actually rarely introduce them.” (Working mother from PM group, bachelor education)*

In line with lower CFPQ in restriction, a mother in the PM group reported that she was still hesitant to try offering a variety of foods and allowed when her children only like the same types of food, such as just rice and fried chicken. On the other hand, a mother from the LB group was more decisive in determining what her child ate and more likely to set limits when the food was unhealthy.

*“My child often finishes his meals only when he has rice and fried chicken at home* **, *so I often end up giving my child the same food repeatedly. I admit I did not*** *try offering other types of food, because he strongly refuses and shuts his mouth.* ***I am afraid of forcing him too much.”*** *(Working mother from PM group, bachelor education)*

“For example, if my child wants to eat pizza today, **I will say that I have already cooked, so just eat what has been prepared**.” *(Working mother from LB group, post graduate education)*

Working mothers observed that at home, children had smaller portions and lower appetites compared to daycare, where they consistently finished their meals and often asked for more. However, staff did not specify portion sizes noting that they varied with the child’s appetite.

*“* ***The appetite tends to decrease a bit at home, and the portion sizes are also smaller compared to daycare*** *.” (Working mother from LB group, post graduate education)*

*“Yes, it depends on the child…* ***Sometimes there are children who should eat this amount, but they are in the mood for more***, *so they ask for extra” (Staff from PM group, high school education)*

Working mothers reported challenges in getting their children to eat at home compared to daycare, where children were more willing to eat, leading to a repetitive home menu. These findings suggest that despite varied daycare menus, limited home options or portions may impact a child’s nutrient intake.

*“… having difficulty eating, from childhood until now, it has been a struggle.* ***Even now, I still do not know how to make them eat properly… except in daycare, where they eat willingly*** *.” (Working mother from LB group, post graduate education)*

During mealtimes, the daycare staff in PM group supervised children to ensure they stay seated and behave during meals, with management overseeing mealtime. These efforts underscore the daycare’s role in assisting with feeding, particularly for children facing eating challenges, highlighting the significant responsibility of both staff and management.

*“* ***We sit beside them*** *… so they sit in a row to eat.* ***They are not allowed to walk around*** *… the teachers walk around, but the children must stay seated and behave.” (Staff from PM group, high school education)*

*“Yes, during meals the daycare staff* ***help to feed and accompany the children…*** *” (Management from PM group, diploma education, 21 years of work).*

Staff observed that children often struggled with eating vegetables, possibly due to limited exposure at home. This was supported by daycare encouragement, where children ate vegetables with their friends, motivating each other. Parents also confirmed that their children found it challenging to eat vegetables at home.

*“…. children usually find it difficult to eat vegetables because* ***they are not accustomed to it at home*** *.” (Staff from PM group, diploma education)*

*“Sometimes, there are parents who wonder, “Why does my child/the child like vegetables at daycare but not at home?” The vegetables at daycare are usually consume first because the communal aspect of eating together with friends is important.* ***Seeing other friends eating encourages them to try. It is quite effective at times.*** *(Staff from PM group, high school education)*

*“However,* ***vegetables are still a bit challenging*** *.” (Working mother from LB group, post graduate education)*

The mothers noted that larger portion sizes and increased appetite during communal lunch sessions at daycare motivated children to finish their meals. They also highlighted the role of peer influence, with friends helping children enjoy their meals.

*“* ***The portion sizes are bigger and appetite of children are higher at daycare*** *because there is a communal lunch session, which motivates children to “compete” in finishing their meals”. (Working mother from LB group, post graduate education)*

### Qualitative: Nutrition knowledge

Some staff and management were familiar with balanced nutrition guidelines, while others had never heard of it.

*“* ***I have heard of that one***, *where there’s a plate with the main dish in the photo.” (Management from LB group, bachelor education, 2 years of work)*

*“* ***Not yet***, *ma’am” (Staff from PM and LB groups)*

The staff and management admitted they had never received any nutrition training or education, although they felt it was important to communicate balanced nutrition messages to parents.

*“* ***I never attended any nutrition training*** *nor nutrition education” (Staff from PM and LB groups)*

*“* ***Neither nutrition education nor nutrition training*** *” (Management from PM group, diploma education, 21 years of work).*

A mother from the LB group mentioned that she sometimes received or seek information about children’s food through social media. Meanwhile, a mother from the PM group stated that she did not use any references when preparing her child’s meals at home.

*“* ***Sometimes I read on Instagram that child should eat this and that***. *I try to follow all the information. Then I realized, oh, there are specific portion sizes that should be met.” (Mother from LB group, post graduate education)*

*“When preparing meal plans,* ***I do not use any references. I just make decisions on my own*** *.” (Mother from PM group)*

## Discussion

The findings of the study reveal an interesting contrast between children in PM group and LB group. Even though dietary diversity was better in PM group, nutrient intakes were lower among children in the PM group compared to those in the LB group. Moreover, children in PM group had more inadequate nutrient intakes particularly for calcium and folate compared to those in the LB group. The qualitative findings found more diverse foods and more favourable eating environment in PM group, but at the same time also more skipping breakfast. The staff and management at daycare, even in the PM group, had never received any training on nutrition education. Our finding showed that child feeding practices of the working mothers in PM group were lower in supporting environment, encouragement of balance and variety, and restriction. This suggested that the food provision had not been accompanied by effort to also strengthen mothers’ feeding practices.

The higher dietary diversity among children in the PM group may be attributable to the efforts of daycare providers to create menus with a variety of options for the children. The qualitative finding supported that the daycare management had already made efforts to create varied menus. This was also confirmed by working mothers that the menu provided by the daycare was already very diverse, and the teachers documented it in the communication book. This aligns with similar research on children receiving School Feeding Programmes (SFP) and those who do not, where DDS among SFP beneficiaries is higher than non-SFP beneficiaries.^(32)^

Another important aspect that warrants attention is the conducive eating environment in the daycare setting, which has fostered children’s habituation to vegetable consumption within daycare more noticeably than at home. The qualitative findings from daycare staff and management also indicated that children were more willing to eat vegetables at daycare, as the communal eating environment positively influenced their eating behaviour. In contrast, parents reported that their children were often reluctant to eat vegetables at home. This finding aligns with research conducted in Oklahoma, which found that children consumed more fruits and vegetables at daycare than at home.^(33)^ Moreover, the portion size of children’s meals at daycare is larger compared to when they eat at home. Qualitative findings from working mothers indicated that children received smaller portions at home compared to daycare, with home portion sizes not meeting the target. Previous studies indicate that working mothers’ decisions about what and how much to feed their children are influenced by the child’s food preferences, time limitations, and challenges in meal planning.^(30–31)^ These aspects are important to incorporate in nutrition-related information for parents, so that they can manage their children’s eating at home.

Although the PM group had better dietary diversity than the LB group, their overall nutrient intake was lower. In particular, children in the PM group showed higher inadequacy in calcium and folate intake compared to those in the LB group. This was attributable to the common habit of skipping breakfast among children in the PM group. Daycares providing meals did not address this issue when children arrived without having had breakfast. Qualitative findings from staff and management confirmed that children often came to daycare hungry, as they had not had breakfast and the daycare neither provided breakfast nor allowed packed meals. Children had to wait until lunchtime or morning snack time. Parents also reported that their children frequently skipped breakfast due to time constraints and lack of morning appetite. At that time, daycare managers and staffs were uninformed of what should be done to ensure that children did not have to wait until lunch time. Previous study stated that missing healthy breakfasts, which are usually rich in nutrients like calcium and fibre, results in lost opportunities to provide essential energy and nutrients necessary for growth and healthy development.^(36)^ Another previous study also reported that children who skipped breakfast had a higher prevalence of folate and calcium inadequacy compared to those who ate breakfast.^(37)^ This finding was supported by the absence of nutrition education ever received by daycare staff and management.

Another factor that can contribute to the lower nutrient intake in PM group is that the scores of working mothers in the LB group were higher than those in the PM group in the categories of environment, encouragement of balance and variety, and restriction. The higher scores among working mothers in the LB group indicated that greater attention was given to the environment when feeding their children, encouragement of variety and balance in their diet, and better regulation of their children’s eating patterns compared to working mothers in the PM group. This difference may be attributed to the higher level of education among mothers in the LB group, which could influence their awareness in planning nutritious lunchboxes for their children. On the other hand, working mothers in PM group had lower scores in supporting environment, encouragement of balance and variety, and restriction, which might be due to over-reliance to daycare to provide the foods for their children. The qualitative findings revealed that mothers in the LB group often search for information on social media related to nutritious foods and appropriate portion sizes for their children’s meals.

Our findings were in contrast with study conducted in the Netherlands, which reported higher energy and carbohydrate intake among children attending daycare as compared to those who ate at home.^(38)^ The discrepancy may be attributable to differences in study contexts, as the previous study was conducted in developed country and compared daycare meals with home meals. In addition, variations in national food-based dietary guidelines, particularly for young children, applied in the Netherlands versus in Indonesia may have also contributed to these differing results. The findings from the nutrient intakes of the children, feeding practices of the working mothers, and perceptions from mothers, daycare staff and management had implications for strengthening the guideline for food provision at daycare as well as highlighting the importance of nutrition education for daycare staff and management, and ensuring adequate nutrition-related information for the working mothers. For daycares that provide meals, it is crucial to educate parents on the necessity of not skipping breakfast. In addition, daycares should implement strategies to prevent children from arriving hungry and to manage or mitigate breakfast skipping. Strengthening Standard Operating Procedures (SOPs) related to the provision of breakfast or having standby meals available is essential. For daycares where children bring lunchboxes, it is equally important to provide education for parents, particularly encouraging children to enjoy eating vegetables at home and promoting dietary diversity. The findings of this study support the adjustment of the ECCNE model in daycare settings, which include the promotion of food-based recommendations, nutrient-dense menu for food provision in daycare, and nutrition education for staff and management. Providing nutrition-related information for working mothers remain critical in either condition, whether foods are provided at daycare or brought from home.

### Strengths and limitations

To our knowledge, no prior study has compared the nutrient intakes of children consuming daycare-provided meals versus those who brought food from home, while simultaneously exploring the perspectives from working mothers, daycare staff, and daycare managers regarding the provision of nutritious meals. The dietary assessment method utilised in our study combined weighed records and estimated food records, which adds accuracy to the nutrient intake estimates. This study represented the first attempt to compare child feeding practices among working mothers and daycare staff in Indonesia, particularly within daycare facilities under governmental ministries/agencies. Furthermore, our study reported child feeding practices among working mothers to have better understanding of the child feeding aspects that need to be strengthened.

However, the study had limitations, including a relatively small sample and the difference in maternal education background between the PM and LB, which may have partly confounded the finding. Since daycares involved in the studies were those in government ministries, they may not be representative of daycares in other settings e.g. community-based daycares.

## Conclusions

This study showed that children in the PM group have higher dietary diversity amongst 6–23 months children, but lower nutrient intake and higher proportions of inadequate nutrient intakes than LB group. The child feeding practices reported by working mothers and the qualitative data on the views of working mothers, daycare staff, and daycare managers regarding child feeding practices and food provision, emphasises the need for nutrition education for both working mothers and daycare staff to improve attitudes and awareness regarding breakfast consumption, menu planning, and portion sizes. Further study is needed to develop food-based recommendations and nutrient-dense menu for food provision in daycare; to design package of nutrition education for daycare staff and management and nutrition-related information for working mothers; and to test the effectiveness of the intervention in improving mothers’ feeding practices, nutrition knowledge and food provision at daycare, and eventually dietary diversity and nutrient intakes of children in daycares.
